# Age-Dependent Immune Defense Against *Beauveria bassiana* in Long- and Short-Lived *Drosophila* Populations

**DOI:** 10.3390/jof11080556

**Published:** 2025-07-27

**Authors:** Elnaz Bagheri, Han Yin, Arnie Lynn C. Bengo, Kshama Ekanath Rai, Taryn Conyers, Robert Courville, Mansour Abdoli, Molly K. Burke, Parvin Shahrestani

**Affiliations:** 1 Department of Biological Science, California State University Fullerton, 800 N State College Blvd, Fullerton, CA 92835, USA; 2 Department of Mathematics, California State University Fullerton, 800 N State College Blvd, Fullerton, CA 92835, USA; 3 Department of Integrative Biology, Oregon State University, Cordley Hall, 3029, 2701 SW Campus Way, Corvallis, OR 97331, USA

**Keywords:** entomopathogenic fungi, arthropod immunity, host-pathogen interactions, aging

## Abstract

Aging in sexually reproducing organisms is shaped by the declining force of natural selection after reproduction begins. In *Drosophila melanogaster*, experimental evolution shows that altering the age of reproduction shifts the timing of aging. Using the Drosophila experimental evolution population (DEEP) resource, which includes long- and short- lived populations evolved under distinct reproductive schedules, we investigated how immune defense against *Beauveria bassiana* changes with age and evolved lifespan. We tested survival post-infection at multiple ages and examined genomic differentiation for immune-related genes. Both population types showed age-related declines in immune defense. Long-lived populations consistently exhibited age-specific defense when both long- and short-lived populations were tested. Genomic comparisons revealed thousands of differentiated loci, yet no enrichment for canonical immune genes or overlap with gene sets from studies of direct selection for immunity. These results suggest that enhanced immune defense can evolve alongside extended lifespan, likely via general physiological robustness rather than traditional immune pathways. A more detailed analysis may reveal that selection for lifespan favors tolerance-based mechanisms that reduce infection damage without triggering immune activation, in contrast to direct selection for resistance. Our findings demonstrate the utility of experimentally evolved populations for dissecting the genetic architecture of aging and immune defense to inform strategies to mitigate age-related costs associated with immune activation.

## 1. Introduction

In evolutionary biology, aging is defined as a decline in adaptive capacity caused by the weakening of natural selection after the age of first reproduction in sexually reproducing populations [1,2,3,4,5]. This definition is supported by a robust body of experimental evidence demonstrating that the timing of aging can be altered through laboratory selection on reproductive onset [2,6,7].

The Drosophila experimental evolution population (DEEP) resource, commonly referred to as the Rose population, includes the largest and most extensively studied collection of laboratory-evolved *Drosophila melanogaster* populations, e.g., [2]. This resource includes populations that have undergone direct selection on reproductive onset and become differentiated in life history traits, including lifespan [8]. Responses to such selection are repeatable, resistant to evolutionary history, consistent across replicate populations, and accompanied by widespread allele frequency shifts across the genome [8,9]. Comparisons between long- and short-lived populations of the DEEP resource have revealed enhanced early-life immune defense in long-lived populations [10], and an overrepresentation of immunity-related genes among genomic differences between these populations [11]. Similarly, genome-wide association studies and laboratory selection studies in other Drosophila systems have identified immunity genes as overrepresented among those associated with survival to extreme old age [12,13].

Despite these associations, immune function in *D. melanogaster* is known to decline with age [14,15,16,17], and experimental evolution studies have shown that improvements in pathogen resistance come at the cost of reduced lifespan in uninfected conditions [18]. These findings suggest an evolutionary tradeoff between longevity and immune defense, potentially driven by energetic or resource allocation constraints, and likely impacted by cumulative exposure to microbes throughout the lifespan. However, the universality of this tradeoff remains uncertain.

A common limitation of studies on immune aging in *D. melanogaster* is the confounding effect of cumulative microbial exposure in comparisons between young and old individuals from the same population [19]. Alternatively, comparisons of immunity in mutant lines that differ in aging [20] often involve large-effect alleles that can distort other life-history traits and may not reflect the genetic variation that segregates in natural populations [21].

The DEEP resource provides a unique opportunity to disentangle biological aging from chronological aging. Populations selected for delayed reproduction exhibit extended lifespan and delayed physiological decline, as evidenced by both population-level mortality and individual-level declines in traits like reproductive output and locomotion [22,23,24,25]. Thus, the DEEP resource allows for direct comparisons of flies that are the same chronological age but differ substantially in biological age.

In this study, we used the DEEP resource to investigate the relationship between aging and immune defense using the fungal entomopathogen *Beauveria bassiana*, a widespread natural pathogen of a broad range of insect hosts, including *D. melanogaster. B. bassiana* is environmentally persistent and can be used in biological pest control [26,27,28,29], representing a natural, ecologically relevant challenge that engages both barrier defenses and systemic immune responses in *D. melanogaster* [30,31]. Our objective was to understand how aging and evolutionary history shape immune defense in populations experimentally selected for divergent lifespans. Specifically, we addressed three central questions: How does chronological age affect immune defense in long- and short-lived *D. melanogaster* populations? What is the relationship between evolved lifespan and immune defense in these populations? And, does experimental evolution for lifespan divergence lead to genomic differentiation in immune-related genes?

## 2. Materials and Methods

### 2.1. Drosophila melanogaster Populations

**Figure 1:**
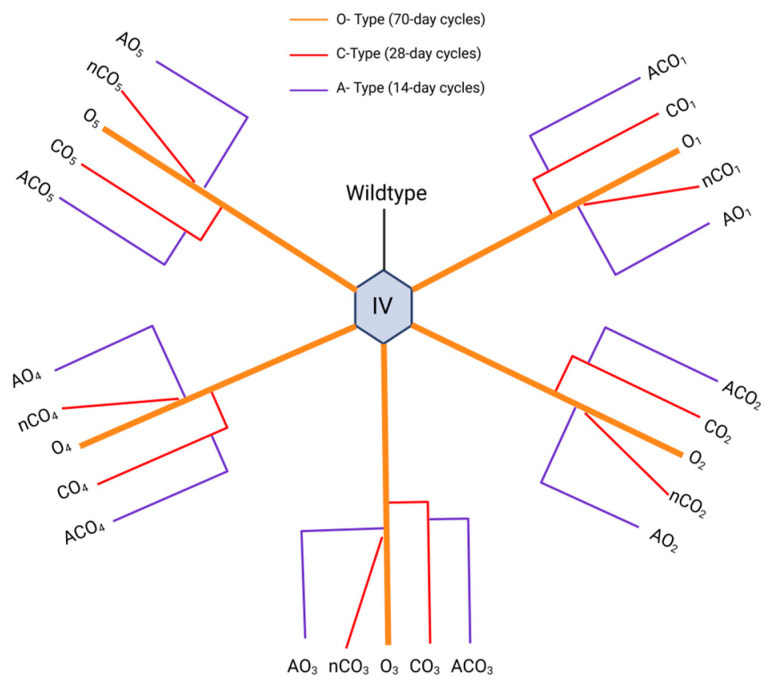
Evolutionary history of the Drosophila experimental evolution population (DEEP) resource used in this study. The ancestral population (IV) originated from an apple orchard in 1970. Five “O-type” populations (orange) were derived from the IV and selected for delayed reproduction on 70-day discrete generation cycles. The C-type (CO and nCO) populations (red) emerged from the O-type and were selected on 28-day discrete generation cycles. The A-type (ACO and AO) populations (blue) were selected for early age of reproduction, on 10-day generation cycles.

We used populations from the DEEP resource, all derived from the ancestral IV population collected from an apple orchard in Massachusetts (Figure 1). The IV population was originally maintained on 14-day discrete generation cycles (from egg to egg). In 1980, five replicate populations (O_1–5_) were created by gradually extending generation cycles to 70 days [6]. In this system, replicate populations are populations that undergo the same selection regime but are genetically isolated from each other. From these, five replicate populations (CO_1–5_) were derived and maintained on 28-day cycles [32]—subscripts indicate shared lineage, for example, CO_1_ was derived from O_1_. Another five populations (ACO_1–5_) were derived from the CO lines and selected for 10-day generation cycles [7].

The ACO and CO populations have undergone hundreds of generations of divergent selection and differ significantly in phenotypes, including lifespan, development time, reproductive output, stress resistance, and activity levels [8,26,33]. These populations also show genome-wide divergence in allele frequencies [9,11].

For phenotyping immune defense, we used four replicates each of the ACO (ACO_1,3–5_) and CO (CO_1,3–5_) populations. For genomic analyses, we expanded the sample to include 20 populations in total: 10 short-lived (ACO_1–5_, AO_1–5_) and 10 long-lived (CO_1–5_ and nCO_1–5_). These additional populations share the same selection regimes and have converged in genome-wide allele frequencies with their respective population types [9].

During stock maintenance and testing, all populations were maintained in the same environmental and dietary conditions they had been kept in for hundreds of generations since domestication, with a controlled diet, temperature, and light [2]. Specifically, the populations were maintained on a banana-molasses diet at 25 °C under constant light. The diet composition per liter of distilled water was: 13.5 g Apex Drosophila agar type II (Genesee Scientific, El Cajon, CA, USA), 121 g peeled ripe banana, 10.8 mL each of Light and Dark Karo syrup (ACH Foodservice, Oakbrook Terrace, IL, USA), 16.1 mL Eden organic barley malt syrup (Eden Foods, Clinton, MI, USA), 32.3 g active dry yeast (Lesaffre Yeast Corporation, Milwaukee, WI, USA), 2.1 g methyl-4-hydroxybenzoate as anti-fungal (Sigma-Aldrich, St. Louis, MO, USA) and 42.5 mL ethanol. Nutritional content per liter was approximately 1.2 g fat, 37.2 g total sugar, 90.4 g total carbohydrates, 21.2 g protein, and 450 kcal [34].

### 2.2. Immune Defense Assay

Before immune testing, ACO_(1,3–5)_ and CO_(1,3–5)_ populations (four biological replicates) were reared for two generations on a common 14-day cycle to minimize environmental and parental effects. On day 13 from egg laying, adults were transferred to plexiglass cages at densities of ~1500–2000 flies per cage.

Flies were tested for immune defense every two weeks throughout their lifespan. Specifically, the ACOs were tested at ages 14, 28, and 42 days post-egg, and the Cos were tested at ages 14, 28, 42, 56, and 70 days post-egg. Immune defense was quantified as survival following inoculation with the entomopathogenic fungus *Beauveria bassiana* ARSEF 12460 [31]. Flies were briefly anesthetized using carbon dioxide, placed on ice-chilled Petri dishes, and sprayed using a custom-built spray tower [35,36] with 5 mL of a fungal suspension (0.36 g of spores in 30 mL of 0.03% Silwet L-77 (Momentive Performance Materials, Niskayuna, NY, USA) in DI water), delivering ~10^3^ spores/mm^2^ to the fly cuticle. These methods were aligned with our previous work [18,36].

Inoculated flies were placed into Plexiglass cages (450 cm^3^) at densities of 100–150 flies/cage, sexes mixed and kept at 25 °C and 100% humidity for 24 h to allow the fungus to germinate. In addition to the four-fold biological replication, we included two technical replicates per each test condition (control, uninfected, or infected) for each population at each age. Afterward, cages were kept at 25 °C at ~50% humidity. Uninfected controls were handled identically but sprayed with Silwet solution alone. An additional “untreated” control at age 14 was not anesthetized or sprayed but was otherwise maintained identically to the other test conditions. Mortality was recorded daily for all test conditions. Specifically, dead flies were removed from the cages, sorted by sex, and counted to quantify sex-specific mortality. Sporadically, dead flies from the control, uninfected, and infected groups were checked to see if they had *Beauveria bassiana* in them by looking for *B. bassiana* sporulation on dead flies on SDAY media.

To confirm that spore viability was >90% for all sprays, a 2 mL suspension of a 1:1000 dilution of the suspension was sprayed through the Spray Tower onto a 60 × 15 mm water agar Petri dish, which was incubated at 25 °C for 24 h. Following incubation, one hundred spores within a central swath were inspected under a light microscope for the presence or absence of a growing germ tube. Spores with germ tubes greater than or equal to the length of the spore were tabulated as living, while others were considered non-viable. To estimate the infection dose, a plastic microscope coverslip (22 × 22 mm) was placed adjacent to the flies during the sprays. After each coverslip was dried, it was placed in a 50 mL centrifuge tube with small glass beads and 5 mL of 0.03% Silwet solution and vortexed [37]. Then 10 μL of this spore suspension was placed onto another microscope slide and the number of spores was counted under a light microscope.

### 2.3. Statistical Analysis of Phenotype Data

We analyzed survival data using an exponential Weibull regression model [38], incorporating covariate treatment conditions, age groups, sex (male vs. female), and the interaction terms. The model-generated survival estimates closely approximated the observed survival data for both control flies and uninfected flies of A-type and C-type populations. Bayesian simulations were employed to generate 95% credible intervals around the estimates of the survival function. All analyses were performed in the statistical software R version 3.6). 

To determine changes in immune defense in A-type populations and C-type populations we compared post-infection survival using the exponential Weibull regression model with the Markov chain Monte Carlo (MCMC) sampling method, a Gibbs sampler [39], and the Metropolis–Hastings [40] algorithm to assess the properties of survival function, hazard ratio, and percentage of change on scale parameters as a measurement of immunity.

We assumed the time to death followed an exponentiated Weibull distribution with shape and scale parameters, where the scale parameter was modeled as an exponentiated linear combination of covariate treatment, sex, age groups, and any interaction terms. Using prior distributions and the sampling methods, we simulated 800,000 to 1,000,000 draws from posterior distributions of (α, θ, i′s). The first 200,000 to 300,000 draws were discarded as burn-in. The remaining draws were checked for autocorrelation and thinned by selecting one draw every 200 to 250 draws; at least 2000 draws remained (α, θ, i′s). Using these final samples, we computed estimates and 95% credible intervals of shape parameters, covariate coefficients, survival functions, and hazard ratios.

The simulated data allowed us to calculate the percentage of change in scale parameters as a measurement of immune defense. Holding the shape parameters as constant, the survival function is stretched to the right as the scale parameter increases and compressed toward the initial experiment day as it decreases. We first computed the scale parameters for infected and uninfected groups of the same population at the same age, for example, ACO at age 14. Next, we computed and quantified the percentage of change in scale parameters between the infected and uninfected. If the percent change was sufficiently smaller in one group compared to another, we concluded that the group had stronger immunity. For example, because the value for ACO at age 14 was smaller than at age 28, we concluded that the immunity at age 14 was better than at age 28.

The exponential Weibull regression model also allowed us to assess sexual dimorphism in terms of survival using hazard ratios. For example, we calculated the hazard ratio and 95% credible intervals for infected and uninfected groups at all ages (14, 28, and 42 days). We compared the hazard ratios to one; if the hazard ratio was above 1, the baseline group had a lower hazard and therefore survived better.

Using the Bayesian simulations, we also computed the median residual lifetime and its 95% credible intervals for ACO and CO populations. The median residual lifetime is defined as the time interval from a given starting point t_0_ to the point at which half of the flies alive at time t_0_ are still alive.

This metric provides additional insights into immunity measurement. We also computed the percent of change in scale parameters and their related 95% credible intervals for both populations across all ages. Based on the exponentiated Weibull regression model, these immunity measures enabled us to compare ACO and CO populations of the same age directly.

### 2.4. Meta-Analysis of Candidate SNPs

To test the prediction that genes associated with immune defense might be differentiated between the ACO and CO populations, we mined previously obtained, publicly available sequence data from these populations. Graves et al. [9] identified a list of genome-wide SNPs that were significantly differentiated between populations with A-type (generation time of 9–10 days) and C-type (generation time of 28 days) ancestry. These A-type and C-type populations of Graves et al. [9] include the populations phenotyped in the present study, as well as additional populations that had experienced identical selection conditions (ACO_1–5_, AO_1–5_, CO_1–5_, and nCO_1–5_ populations). We identified all unique genes containing these significant SNPs using the relevant genome release (r5.51). We ran the resulting gene list through LAGO (https://go.princeton.edu/cgi-bin/LAGO, accessed on 15 March 2021; [41]) to search for enriched GO terms exceeding a significance threshold of *p* < 0.01, using a Bonferroni correction. Results were corrected for hierarchical clustering using GO-Module [42].

We also compared this list of genes to two other relevant *D. melanogaster* experimental evolution publications: (i) Fabian et al. [13], which is itself a meta-analysis comparing three different experiments in which *D. melanogaster* populations were selected for postponed ages of reproduction; and (ii) Shahrestani et al. [18], which sequenced *D. melanogaster* populations that had experienced 19 generations of selection for defense against the same strain of *Beauveria bassiana* that we use in this study. Our rationale behind making the first comparison is that if immune defense is a primary trait driving the evolution of aging, then we should observe genes associated with immune defense in populations selected for both accelerated and postponed ages of reproduction. Our rationale behind making the second comparison is that if immune defense is a key trait driving the evolution of aging, then we should observe the same candidate genes in populations selected directly for immune defense as we observe in populations selected for accelerated and/or postponed ages of reproduction. The R package SuperExactTest (version 1.0.7, Bioconductor 3.12, R 4.0.3) was used to compare matches between these three sets of genes; this tool uses a forward algorithm-based procedure to calculate the intersection probability among sets, with the computational complexity linear to the number of sets [43].

## 3. Results

We tested age-specific survival of short- and long-lived *D. melanogaster* under three conditions: fungal-infected (inoculated with fungus), uninfected (inoculated with just the Silwet solution, without fungus), and control (not inoculated at all) (Figure S1). First, we validated that flies in the fungal-infected group were indeed infected by checking for sporulation on dead flies; as expected, sporulation only occurred in dead flies from the fungal infection groups and not in dead flies from the control and uninfected groups. This was aligned with our observation that under otherwise identical conditions, survival was always lower in fungal-inoculated groups.

Comparing survival between uninfected and control conditions revealed that the handling of inoculation did impact survival, but in inconsistent, and likely biologically unimportant ways (Figure S2). Specifically, in the short-lived populations, the uninfected females and males survived better than controls, and in the long-lived populations, the uninfected females survived worse than the controls, while male survival did not differ between uninfected and control groups (Figure S2).

**Figure 2:**
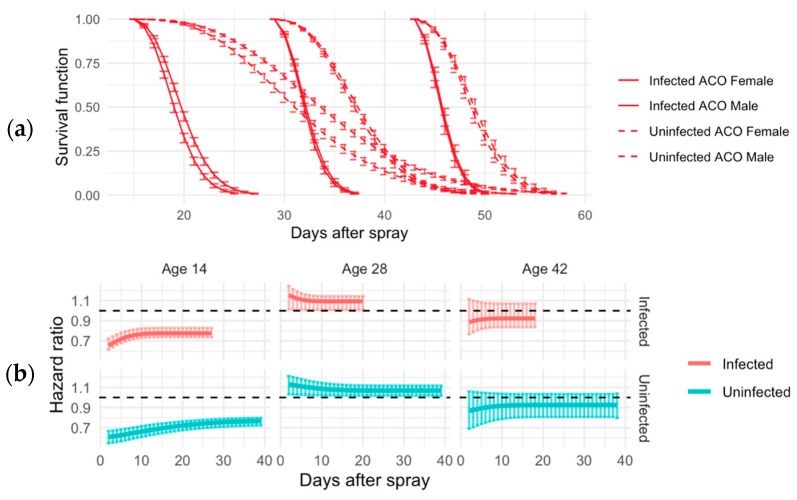
Sex-specific survival patterns and hazard ratios in ACO populations across infection status and age. (**a**) Survival curves for male and female flies in ACO populations, comparing infected (solid lines) and uninfected (dashed lines) groups. Estimates are based on an exponentiated Weibull regression model, which accounts for sexual dimorphism in survival. Vertical lines indicate 95% credible intervals calculated via Bayesian simulations. (**b**) Age-specific hazard ratios (HRs) and 95% credible intervals for infected (red) and uninfected (blue) groups at 14, 28, and 42 days. Males serve as the baseline (black line at HR = 1). HR values above 1 indicate greater survival for males, while values below 1 indicate greater survival for females.

In the short-lived ACO populations, at all ages, infected flies died faster than uninfected flies (Figure 2). Among 14-day-old flies, females survived better than males regardless of infection status (Figure 2). For those sprayed at age 28, 28-day-old flies, males survived better than females regardless of infection status (Figure 2). Finally, flies tested at age 42 demonstrated no observable sex differences in either infected survival or uninfected survival (Figure 2). Upon observing males only, the hazard ratio shows an increased association between infection and reduced survival when sprayed at age 28 compared to ages 14 and 42 (Figure 2).

**Figure 3:**
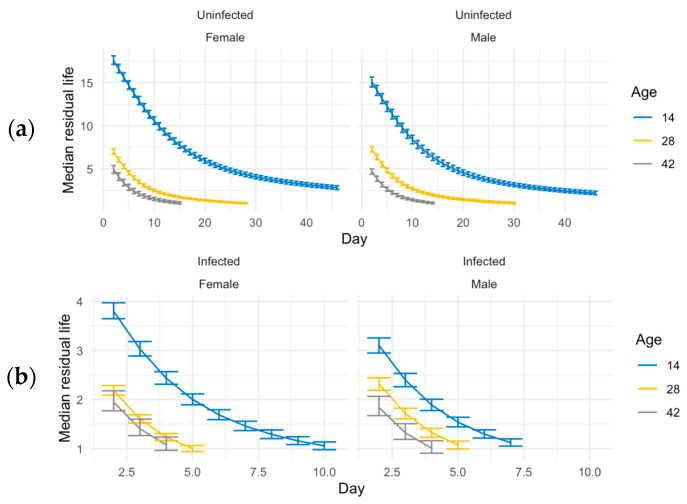
Median residual lifetime estimates for infected and uninfected ACO populations. (**a**) Median residual lifetime for uninfected ACO flies. (**b**) Median residual lifetime for infected ACO flies. In both panels, estimates are shown at 14 days (blue), 28 days (yellow), and 42 days (gray) post-spray. For both panels, median residual lifetime and corresponding 95% credible intervals were estimated using Bayesian simulations. The median residual lifetime represents the expected time from a given point (t_0_) until half of the individuals alive at t_0_ are still alive. This measure offers insight into age-specific survival and may serve as a proxy for immune resilience.

In our study, both infected and uninfected groups are followed until all flies die. Therefore, an estimate of “immune defense” could be made from comparisons of uninfected and infected groups, instead of using just the survival of the infected groups. We used median residual lifetime and related 95% credible intervals for short- and long-lived populations for insights into the immune defense phenotype. We measured the t50 at every age by looking at the residual life from that age in the ACO populations (Figure 3). We calculated the percent of change on scales and related 95% credible intervals to measure immune defense for the ACO populations (Table 1). Younger (14-day-old) ACO flies had better immune defense than older flies; the changes between uninfected and infected groups at age 14 are significantly smaller than they are at ages 28 and 42 (Table 1).

**Figure 4:**
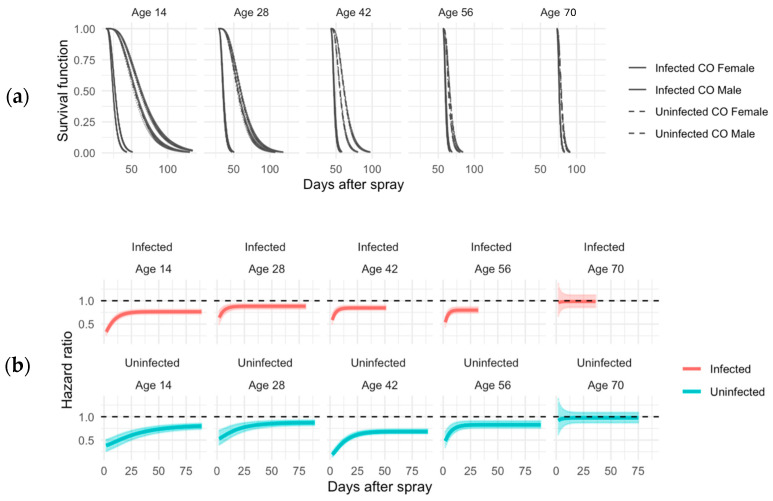
Sex-specific survival and hazard ratios in CO populations across infection status and age. (**a**) Survival curves for male and female flies in CO populations, comparing infected (solid lines) and uninfected (dashed lines) groups. Estimates are derived from an exponentiated Weibull regression model, which accounts for sexual dimorphism in survival. (**b**) Age-specific hazard ratios (HRs) and associated 95% credible intervals for infected (red) and uninfected (blue) groups at 14, 28, 42, 56, and 70 days. Males serve as the baseline (black line at HR = 1). HR values above 1 indicate greater survival for males; values below 1 indicate greater survival for females.

**Figure 5:**
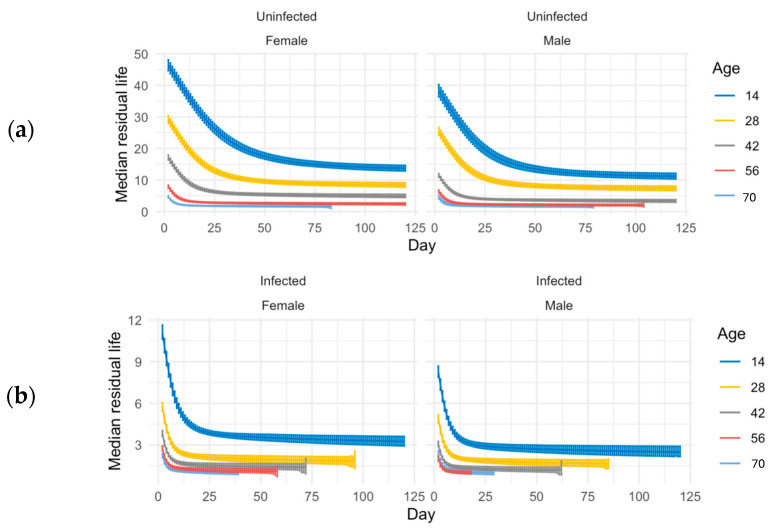
Median residual lifetime estimates for infected and uninfected CO populations. (**a**) Median residual lifetime for uninfected CO flies. (**b**) Median residual lifetime for infected CO flies. In both panels, estimates are shown at 14 days (dark blue), 28 days (yellow), 42 days (gray), 56 days (red), and 70 days (light blue) post-spray. As with Figure 3, the median residual lifetime and corresponding 95% credible intervals were estimated using Bayesian simulations.

Long-lived CO populations were also tested at ages 14, 28, and 42, but these were additionally tested at ages 56 and 70 when the ACO flies had died (Figure 4), and t_50_ was measured for each age (Figure 5). At ages 14, 28, 42, and 56, female CO flies survived better than males in both infected and uninfected conditions (Figure 4). However, there was no sexual dimorphism seen in survival with flies sprayed at age 70 (Figure 4). Younger CO flies had better immune defense than older CO flies (Table 2). In the CO populations, there was also sexual dimorphism in immune defense at ages 14 and 42 with females having better immune defense than males (Table 2).

**Figure 6:**
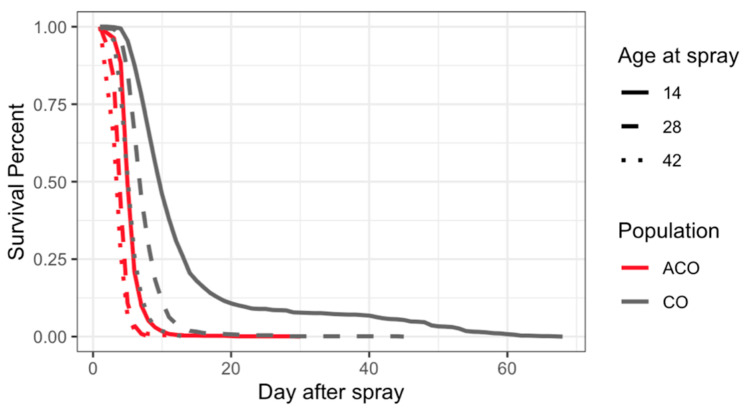
Survival patterns of infected ACO and CO populations across age and time post-treatment. Percent survival over time for infected ACO_(1,3–5)_ (red lines) and CO_(1,3–5)_ (gray lines) populations at three ages: 14 days (solid lines), 28 days (dashed lines), and 42 days (dotted lines). The y-axis represents percent survival, and the x-axis shows days after spray treatment. A visual comparison reveals that the survival trajectory of ACO populations infected at age 14 closely overlaps with that of CO populations infected at age 42, suggesting differences in age-related immune defense between the two groups.

Both short- and long-lived populations were tested at ages 14, 28, and 42, allowing for side-by-side comparisons of infection survival at these ages (Figure 6, Table 3). Immune defense was better in the CO populations compared to the ACO populations at all ages (Table 3).

Graves et al. [9] found 10,109 SNPs differentiated between populations with A-type (i.e., the ACO populations tested here) and C-type (i.e., the CO populations tested here) ancestry; these SNPs were evaluated for significance using Cochran–Mantel–Haenszel (CMH) tests and an empirically determined significance threshold (*p* < 8.21 × 10^−167^). Of these 10,109 SNPs, 8864 were observed in 1136 unique protein-coding genes. The 1136 genes were run through the LAGO GO term enrichment tool, which returned 208 enriched GO terms for biological process at *p* < 0.01. GO-Module further refined this list of GO terms to 33 (Table S1) by removing GO terms that are redundant due to hierarchical clustering. These 33 GO terms generally pertain to organismal development and do not include any related to immune defense. We also did this analysis for just the ACO and CO populations, excluding the AO and nCO (Table S2).

**Figure 7:**
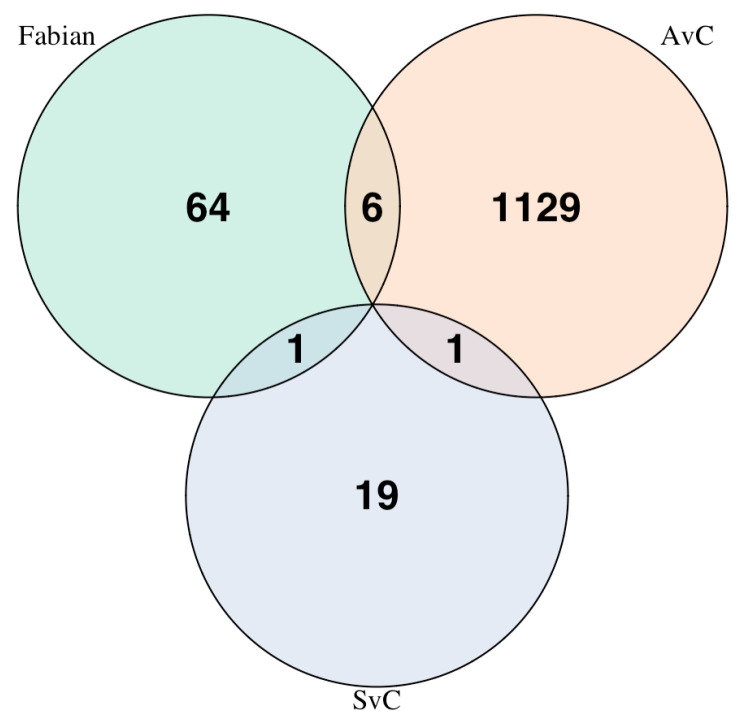
Venn diagram illustrating overlaps between three sets of candidate genes in *Drosophila* evolve-and-resequence experiments. Fabian et al. (2018) identified a total of 71 genes implicated in both selection for postponed reproduction and immune defense [13]. Graves et al. (2017) identified 1136 genes implicated in selection for accelerated development and reproduction [9]. Shahrestani et al. (2021) identified 33 genes implicated in direct selection for immune defense [18]. Only a few genes overlapped across the studies.

We compared the list of 1136 genes differentiating A- and C-type populations to two other relevant gene lists. Fabian et al. [13] reported 71 candidate genes underlying differentiation between populations selected for postponed reproduction and control populations that were also involved in immune response [13]. Shahrestani et al. [18] identified 42 SNPs that were significantly differentiated (using CMH tests and an empirically determined significance threshold of *p* < 3.94 × 10^−18^) between populations selected for defense against a fungal pathogen and controls. Of these 42 significant SNPs, 33 were observed in 21 unique protein-coding genes. Comparing these three gene lists (1136 from Graves et al. [9]; 71 from Fabian et al. [13]; 21 from Shahrestani et al. [18]) returned very few observations of overlap (Figure 7). Fabian et al. [13] had six genes in common with Graves et al. [9] and one gene in common with Shahrestani et al. [18]. Graves et al. [9] and Shahrestani et al. [18] had one gene in common, and no genes were shared by all three studies. Using the R-package SuperExactTest to evaluate multiset intersections, we found no evidence that these overlaps were statistically significant, though the single shared gene between Shahrestani et al. [18] and Fabian et al. [13] was associated with *p* = 0.0809.

## 4. Discussion

This study used experimentally evolved *D. melanogaster* populations to investigate how immune defense changes with age, lifespan, and evolutionary history. By comparing short-lived ACO and long-lived CO populations, diverged under selection for different reproductive schedules, we investigated whether short-lived populations exhibit better immune defense (which would indicate tradeoffs between immunity and longevity), how immunity changes with age in short- and long-lived populations, and whether immune-related genes evolve in parallel with life-history traits. Our findings suggest that immunity has indeed evolved in response to life-history selection but in complex and sometimes counterintuitive ways.

In both ACO and CO populations, immune defense declined with chronological age. Younger flies consistently survived fungal infection better than older flies, demonstrating immunosenescence aligned with prior studies [14,16]. However, the rate of decline differed: CO flies retained higher immune function later in life compared to ACO flies, particularly in females. The faster decline of immune defense in the ACO compared to the CO is consistent with prior findings that physiological decline in the CO populations occurs more slowly than in the ACOs [22,23]. These results support the idea that the pace of immune aging is coupled to the overall rate of biological aging, rather than just to chronological time. While uninfected flies from the ACO also died faster than uninfected flies from the CO, which was consistent with previous results [2], this uninfected mortality rate difference was taken into account in our analyses of mortality of the infected groups. Thus, the differences in the infected groups cannot be explained just by the uninfected mortality differences in these populations.

We observed sexual dimorphism in defense against *B. bassiana*, which corroborates our prior research [18,31,36]. This sexual dimorphism was age-specific, and its age-specificity differed in the ACO and CO populations. While age-specificity in *D. melanogaster* defense against *B. bassiana* has previously been documented [16], our result shows that lifespan evolution can modulate both age- and sex-specific immune defense patterns. Notably, our assays were done on groups of cohabiting *D. melanogaster*. Rai et al. [36] showed that sex-specific immune defense is impacted by mating once and by cohabiting, which potentially allows for multiple matings. The observed impact of lifespan evolution on age-specific sexual dimorphism in immune defense may differ when testing virgin flies.

Across all matched ages (14, 28, and 42 days), the long-lived CO populations exhibited better immune defense than the short-lived ACO populations. As such, populations that evolved for an extended lifespan evolved enhanced immunity at each age tested. Contrary to some expectations of a trade-off between immunity and longevity, e.g., Shahrestani et al. [18], our results show that improved immune defense and increased lifespan can evolve together under long-term selection for delayed reproduction. This pattern may reflect a shared underlying mechanism, such as enhanced overall stress resistance. The CO populations’ extended resilience against fungal infection even in late life suggests that longevity-associated pathways can improve immune robustness, rather than compromise it.

Despite the clear phenotypic differences in immune defense between ACO and CO populations, our analysis of genome-wide SNP data revealed no overrepresentation of immune-related genes among the loci most strongly differentiated between population types. Only minimal overlap was observed between our differentiated genes and previously identified candidate immune genes from studies that directly selected for immunity [18] or longevity [13]. Instead, the most enriched GO terms were associated with developmental and metabolic processes. The overrepresentation of developmental genes was expected given the selection history of the ACO populations, which were directly selected for shortened lifespan by enforcing early reproduction and rapid development. The ACO and CO differ in their developmental time by about three days [8,11].

A growing body of evidence supports the idea that developmental genes can pleiotropically influence adult immune function (reviewed in [44]). However, because canonical immunity genes, such as Toll pathway genes, did not emerge in our analysis, we suggest that the developmental loci identified here may influence adult immune defense via indirect mechanisms. This implies that immune phenotypes may evolve through changes in developmental or regulatory networks rather than through direct selection on classical immune genes. We suggest that selection in these populations has acted on regulatory elements or genes that contribute to general organismal robustness, which incidentally improves immune outcomes.

The distinction between antagonistic immune defense mechanisms (limiting pathogen burden) and cooperative immune defense mechanisms (limiting damage from infection) may help explain why classical immune pathways were not prominent in our genomic data. Selection for delayed reproduction prolongs the impact of natural selection, leading to increased adaptive capacity at later ages. This can select for overall robustness, allowing for increased cooperative responses, often referred to as tolerance [45,46,47,48]. It is also possible that the absence of immune gene enrichment at differentiated loci reflects the complex and polygenic nature of immune defense in outbred populations such as the ones used in this study.

A more detailed analysis of the effects of laboratory selection on immunity may reveal that direct selection for improved immune defense primarily targets antagonistic immune mechanisms, such as pathogen-killing pathways, while selection for delayed reproduction and increased lifespan favors cooperative or tolerance-based mechanisms, which mitigate infection damage without triggering immune activation. This distinction could help explain how long-lived populations maintain stronger immune performance without incurring the cost of systemic inflammation. Recognizing that different classes of genes influence resistance and tolerance may also clarify why some immune phenotypes, like survival after infection, decline with age, while others, such as immune pathway activation, increase with age- reviewed in [19]. These contrasting patterns highlight a promising area for future research into potential biological strategies for mitigating inflammaging and other costs associated with constitutive immune activation.

## Figures and Tables

**Table 1:** Immunity measurement at different ages in ACO populations. Using the Bayesian simulations, we computed the percent of change on scales (estimates) and related 95% credible intervals for ACO populations. These are the changes of scales between the infected and uninfected ACO based on the sigma formula ($\frac{\sigma_{infected}-\sigma_{uninfected}}{\sigma_{uninfected}}$). Sigma is called the scale parameter. Intervals of 95% at age 14 are smaller than at ages 28 and 42. If the quantity is small, we say the change between the groups is small.

Age	Sex	Estimates	95% Intervals
Age 14	Female	0.016	(0.015, 0.017)
	Male	0.019	(0.018, 0.020)
Age 28	Female	0.054	(0.048, 0.059)
	Male	0.052	(0.047, 0.058)
Age 42	Female	0.084	(0.076, 0.097)
	Male	0.089	(0.081, 0.101)

**Table 2:** Immunity measurement at different ages in CO populations. Using the Bayesian simulations, we computed the percent of change on scales (estimates) and related 95% credible intervals for CO populations. These are the changes of scales between the infected and uninfected CO based on the sigma formula ($\frac{\sigma_{infected}-\sigma_{uninfected}}{\sigma_{uninfected}}$). Sigma is called the scale parameter. The table shows 95% credible intervals at ages 14, 28, 42, 56, and 70. If the quantity is small, we say the change between the groups is small.

Age	Sex	Estimates	95% Intervals
Age 14	Female	0.011	(0.010, 0.012)
	Male	0.012	(0.011, 0.013)
Age 28	Female	0.015	(0.014, 0.017)
	Male	0.017	(0.016, 0.019)
Age 42	Female	0.031	(0.028, 0.034)
	Male	0.051	(0.046, 0.060)
Age 56	Female	0.091	(0.082, 0.104)
	Male	0.099	(0.080, 0.112)
Age 70	Female	0.177	(0.159, 0.203)
	Male	0.192	(0.161, 0.225)

**Table 3:** Immunity in ACO versus CO populations. We build an exponential Weibull regression model per age using the Bayesian simulations to find the immunity measurements (estimates) and 95% credible intervals to compare ACO and CO populations of the same age.

Age	Sex	Population	Estimates	95% Intervals
14	Female	ACO	0.041	(0.035, 0.048)
		CO	0.014	(0.013, 0.014)
	Male	ACO	0.063	(0.054, 0.075)
		CO	0.017	(0.014, 0.020)
28	Female	ACO	0.062	(0.055, 0.069)
		CO	0.009	(0.008, 0.009)
	Male	ACO	0.047	(0.042, 0.053)
		CO	0.010	(0.009, 0.011)
42	Female	ACO	0.076	(0.061, 0.096)
		CO	0.016	(0.014, 0.018)
	Male	ACO	0.102	(0.084, 0.126)
		CO	0.024	(0.021, 0.028)

## Data Availability

The original contributions presented in this study are included in the article and Supplementary Materials. Further inquiries can be directed to the corresponding author.

## Supplementary Materials

The following supporting information can be downloaded at: https://www.mdpi.com/article/10.3390/jof11080556/s1, Figure S1: Survival of control, uninfected, and infected ACO; Figure S2: Control versus uninfected survival and hazard ratio; Table S1: GO Term Enrichment in the A versus C-type Populations. 10,109 SNPs were identified as having significantly different frequencies in these two population types. 8864 of these SNPs are in annotated genes. These gene lists were run through LAGO to identify GO term enrichments (at a threshold of *p* < 0.01). 33 GO terms were identified after filtering for hierarchical clustering; Table S2: GO Term Enrichment in the ACO versus CO Populations. 421 SNPs were identified as having significantly different frequencies in these two population types. 321 of these SNPs are in annotated genes. These gene lists were run through LAGO to identify GO term enrichments (at a threshold of *p* < 0.01). 20 GO terms were identified after filtering for hierarchical clustering.

## Abbreviation

The following abbreviation is used in this manuscript:

DEEP	Drosophila Experimental Evolution Population Resource
