# Supplementary material for: Age-Dependent Immune Defense Against Beauveria bassiana in Long- and Short-Lived Drosophila Populations
Source: J Fungi (Basel). 2025 Jul 27;11(8):556. doi: 10.3390/jof11080556 (PMC12387925; doi:10.3390/jof11080556)
Supplement: Supplementary file 1 [file jof-11-00556-s001.zip › jof-3706625-supplementary.pdf]

Supplementary Materials: Figures S1–S2, and Tables S1–S2.

(a)

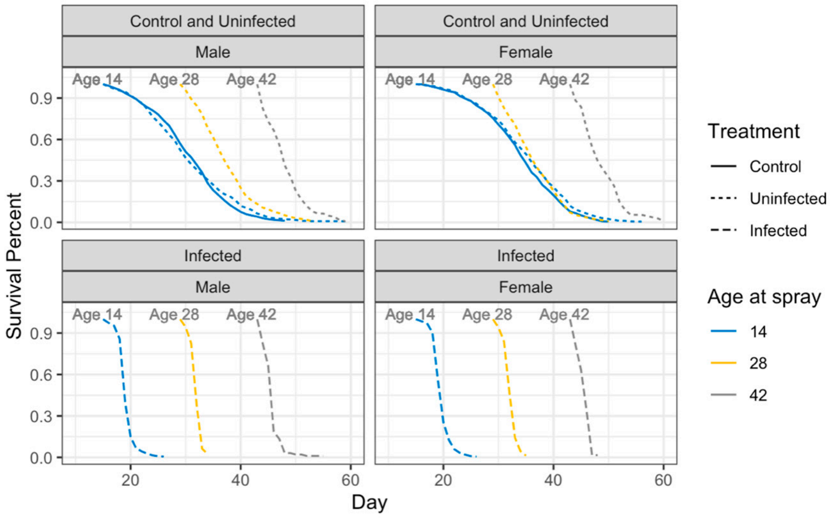

(b)

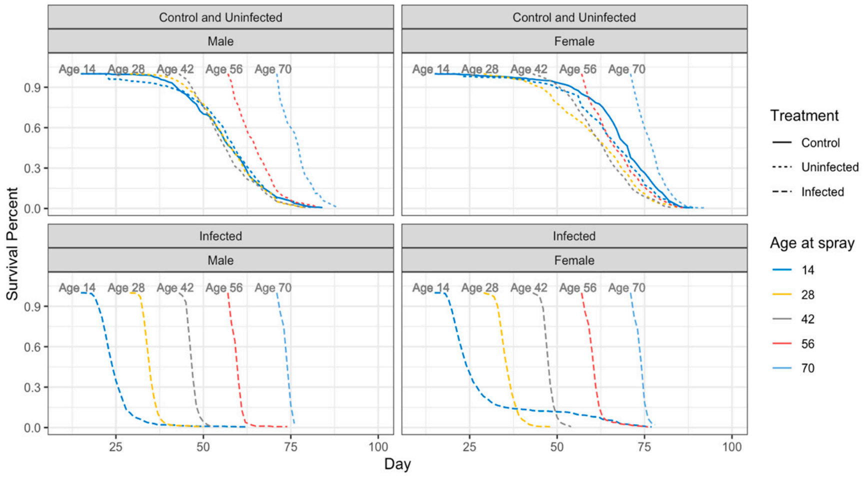

Figure S1. Survival of control, uninfected, and infected ACO. Comparison of survival percent of control (solid lines), un-infected (dot lines), and infected (dashed lines) $ACO_{1,3-5}$ flies at age 14 (blue), 28 (yellow), and 42 (gray) days are shown in both **(a)** male flies and **(b)** female flies. Y axis shows survival percent and x axis show age from egg. The observed survival percent on day t is calculated by 1 minus the cumulative death count till day t divided by the total number of flies. Survival of control, uninfected, and infected CO. Comparison of survival percent of control (solid lines), un-infected (dot lines), and infected (dashed lines) $CO_{1,3-5}$ flies at age 14 (dark blue), 28 (yellow), 42 (gray), 56 (red), and 70 (light blue) days are shown in both panels A (male flies) and B (female flies). Y axis shows survival percent and x axis show age from egg. The observed survival percent on day t is calculated by 1 minus the cumulative death count till day t divided by the total number of flies.

(a)

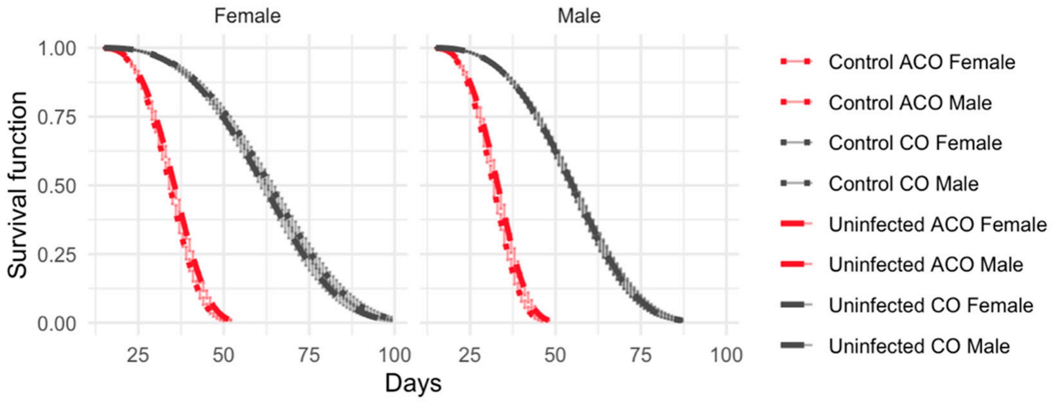

(b)

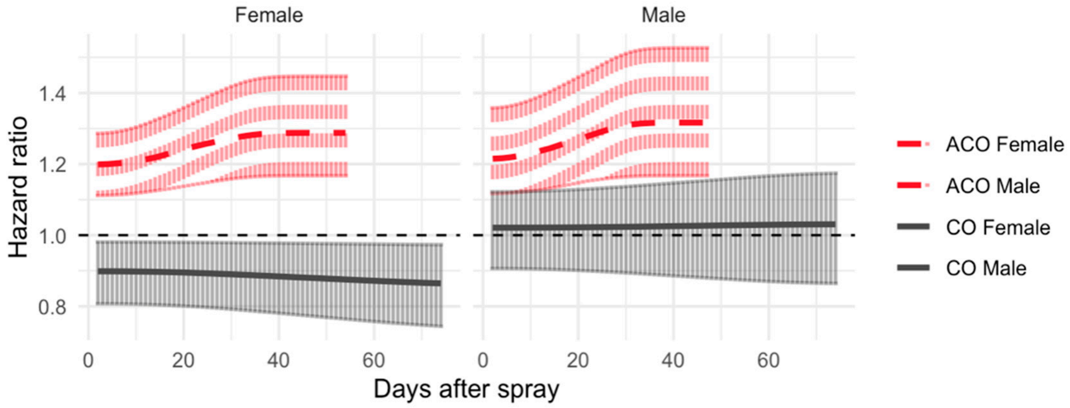

Figure S2. Control versus uninfected survival and hazard ratio. This figure shows survival percent in control and uninfected groups at age 14. Panel A is the model plot. **(a)** The gray color shows comparison of control CO (dot) and uninfected CO (square) groups and the red color displays comparison of control ACO (dot) and un-infected ACO (square). **(b)** The baseline (black line) is the un-infected group. The other groups are compared to the baseline which is set to 1. If hazard ratio above 1, the baseline has less hazard and survives better. ACO un-infected females and males survive better than controls, while CO control females survive better than un-infected females. Results from CO males does not show any significant difference between control and un-infected flies

Table S1. GO Term Enrichment in the A versus C-type Populations. 10,109 SNPs were identified as having significantly different frequencies in these two population types. 8864 of these SNPs are in annotated genes. These gene lists were run through LAGO to identify GO term enrichments (at a threshold of $p < 0.01$). 33 GO terms were identified after filtering for hierarchical clustering.

GO term ID	p-value from LAGO	GO terms
GO:0065007	4.39E-33	biological regulation
GO:0009987	1.90E-29	cellular process
GO:0032501	5.71E-29	multicellular organismal process
GO:0050896	1.90E-26	response to stimulus
GO:0048856	1.77E-23	anatomical structure development
GO:0023052	7.42E-23	signaling
GO:0030154	9.37E-18	cell differentiation
GO:0051179	2.30E-15	localization
GO:0040011	7.28E-14	locomotion
GO:0002009	1.68E-08	morphogenesis of an epithelium
GO:0016477	2.35E-07	cell migration
GO:0061564	4.51E-07	axon development
GO:0048737	5.39E-07	imaginal disc-derived appendage development
GO:0035023	6.10E-07	regulation of Rho protein signal transduction
GO:0048707	6.87E-07	instar larval or pupal morphogenesis
GO:0035114	7.00E-07	imaginal disc-derived appendage morphogenesis
GO:0035120	7.15E-07	post-embryonic appendage morphogenesis
GO:0030707	2.39E-06	ovarian follicle cell development
GO:0007276	3.22E-06	gamete generation
GO:0007610	4.44E-06	behavior
GO:0007409	7.01E-06	axonogenesis
GO:0040007	7.36E-06	growth
GO:0007010	1.94E-05	cytoskeleton organization
GO:0071704	6.18E-05	organic substance metabolic process
GO:0007268	9.27E-05	chemical synaptic transmission
GO:0080090	0.000195333	regulation of primary metabolic process

GO:0051171	0.000223013	regulation of nitrogen compound metabolic process
GO:0060255	0.000462141	regulation of macromolecule metabolic process
GO:0007266	0.000554415	Rho protein signal transduction
GO:0050808	0.00064521	synapse organization
GO:0006468	0.000675618	protein phosphorylation
GO:0044093	0.00135573	positive regulation of molecular function
GO:0001654	0.00172938	eye development

Table S2. GO Term Enrichment in the ACO versus CO Populations. 421 SNPs were identified as having significantly different frequencies in these two population types. 321 of these SNPs are in annotated genes. These gene lists were run through LAGO to identify GO term enrichments (at a threshold of $p < 0.01$). 20 GO terms were identified after filtering for hierarchical clustering.

GO term ID	p-value	GO terms
GO:0009719	5.12E-06	response to endogenous stimulus
GO:0007167	8.54E-06	enzyme linked receptor protein signaling pathway
GO:0009987	2.09E-05	cellular process
GO:0010033	2.30E-05	response to organic substance
GO:0030154	3.89E-05	cell differentiation
GO:0009653	0.000206371	anatomical structure morphogenesis
GO:0023052	0.000211213	signaling
GO:0065007	0.000213405	biological regulation
GO:0046486	0.000221855	glycerolipid metabolic process
GO:0007423	0.00024867	sensory organ development
GO:0009966	0.000274836	regulation of signal transduction
GO:0048477	0.000470947	oogenesis
GO:0046530	0.000581348	photoreceptor cell differentiation
GO:0048749	0.00103327	compound eye development
GO:0001752	0.0013416	compound eye photoreceptor fate commitment
GO:0001745	0.00138436	compound eye morphogenesis
GO:0150063	0.00171913	visual system development
GO:0051704	0.0036784	multi-organism process
GO:0006796	0.00390046	phosphate-containing compound metabolic process
GO:0071840	0.0093236	cellular component organization or biogenesis
